# Computer-Vision-Based Indexes for Analyzing Broiler Response to Rearing Environment: A Proof of Concept

**DOI:** 10.3390/ani12070846

**Published:** 2022-03-28

**Authors:** Juliana Maria Massari, Daniella Jorge de Moura, Irenilza de Alencar Nääs, Danilo Florentino Pereira, Tatiane Branco

**Affiliations:** 1 College of Agricultural Engineering, State University of Campinas, 501 Candido Rondon Avenue, Campinas, São Paulo 13083-875, Brazil; jujumassari@hotmail.com (J.M.M.); djmoura@unicamp.br (D.J.d.M.); tatibranco91@gmail.com (T.B.); 2 Graduate Program in Production Engineering, Universidade Paulista, 1212 Dr. Bacelar Street, São Paulo 04026-002, Brazil; 3 Department of Management, Development and Technology, School of Science and Engineering, São Paulo State University, 780 Domingos da Costa Lopes Avenue, Tupã, São Paulo 17602-496, Brazil; danilo.florentino@unesp.br

**Keywords:** walking ability, animal welfare, animal behavior, image analysis, precision livestock

## Abstract

**Simple Summary:**

We tested two computer-vision-based indexes to analyze the rearing-environment enrichment on broiler movement as a function of comfort temperature and heat stress. The results indicated that the simultaneous application of cluster and unrest indexes could monitor the movement of the group of broilers under different environmental conditions. Future monitoring and alert systems based on computer vision should consider the complexity of the environment for detecting heat stress in broiler production.

**Abstract:**

Computer-vision systems for herd detection and monitoring are increasingly present in precision livestock. This technology provides insights into how environmental variations affect the group’s movement pattern. We hypothesize that the cluster and unrest indexes based on computer vision (CV) can simultaneously assess the movement variation of reared broilers under different environmental conditions. The present study is a proof of principle and was carried out with twenty broilers (commercial strain Cobb^®^), housed in a controlled-environment chamber. The birds were divided into two groups, one housed in an enriched environment and the control. Both groups were subjected to thermal comfort conditions and heat stress. Image analysis of individual or group behavior is the basis for generating animal-monitoring indexes, capable of creating real-time alert systems, predicting welfare, health, environment, and production status. The results obtained in the experiment in a controlled environment allowed the validation of the simultaneous application of cluster and unrest indexes by monitoring the movement of the group of broilers under different environmental conditions. Observational results also suggest that research in more significant proportions should be carried out to evaluate the potential positive impact of environmental enrichment in poultry production. The complexity of the environment is a factor to be considered in creating alert systems for detecting heat stress in broiler production. In large groups, birds’ movement and grouping patterns may differ; therefore, the CV system and indices will need to be recalibrated.

## 1. Introduction

Prospects of future scenarios indicate that the world population will grow to 9.3 billion people in 2050, which requires a significant increase in food demand. Globally, chicken meat is expected to represent 41% of all animal-protein sources by 2030 [1]. In order to meet this strong demand for market growth, intensive production of broilers has prevailed. In most of these systems, the rearing environment restricts opportunities for species-specific behaviors, which are essential for good welfare [2]. Broilers are housed at a high density and with selected genetic characteristics for rapid growth [3]. However, the bone structure of the chicken did not follow this process of high development of the upper body part (breast), which triggers leg disorders and the consequent loss of mobility with increasing body weight [4]. 

The automatic detection of the activity level of groups of broilers makes it possible to identify deviations outside the expected patterns and generate real-time notification alerts to the producer, which allows a faster readjustment with benefits for the welfare of the animals [5]. Stimulating physical activity in birds prevents the occurrence of locomotor problems that impair wellbeing [6,7]. Previous studies indicate that enriched environments have the positive potential to stimulate and increase the activity level of broilers [4,8,9,10,11,12]. Physical activity strengthens the locomotor system, especially at the beginning of the growth phase [13]. In summary, environmental enrichment introduces improvements in existing production systems and considers which artifacts stimulate the behavioral activities inherent to the species, promoting improvements in biological function [14,15]. 

Computer vision (CV) applies mathematics and computer science to provide image-based automated process control [16]. CV allows continuous and real-time measurements during the flock production cycle in a fully automated, noninvasive way [17]. The data images were collected past steps to preprocessing, segmentation (region of interest), features extraction, and classification or regression [16]. Thus, the producer can monitor various biological processes and bioresponses related to animal welfare, health, feeding and drinking behaviors, and flock productivity [18,19,20]. We hypothesize that computer vision associated with movement indexes can monitor locomotor-health problems and prevalence in broiler flocks [5,21,22,23,24]. Studies based on proof of concept are present in animal production and evaluate the technical, practical, and financial feasibility of an idea or hypothesis [25,26]. Several studies have been developed to monitor locomotor-health [22,27,28,29] body-mass estimation [30,31]. The effect of environmental enrichment on broiler activity levels, gait assessment, locomotor problems, zootechnical performance, and behavior and wellbeing have been previously studied using video-image-processing techniques [32,33,34,35]. The computer-vision technology was also validated in a laboratory scale for automatic monitoring and gait-score classification [36]. It was also used to identify abnormal deviations in the activity level of commercial birds [5] and for evaluating the occupancy rate of laying hens in compartments with different levels of ammonia concentration [37]. Under heat-stress conditions, there is a significant decrease in growth rate, increased mortality, a compromised immune system, loss of meat quality, behavioral changes, and a decreased level of wellbeing [38,39]. The rapid diagnosis of animals in thermal discomfort is crucial to prevent the stress from being prolonged, preserving broiler performance, health, and welfare. 

The continuous analysis of image processing obtained by video cameras allows for the generation of activity indexes that monitor the thermal state of broilers [40,41,42]. Most studies in the current literature involving heat stress were conducted from 21 days of age [43]. Animals change their behavioral pattern as a function of the rearing temperature, being close to each other when subjected to cold or spread out in the environment in the heat [42]. Livestock workers have routinely used these postural patterns to assess thermal comfort and adjust to environmental management settings [44]. Thus, observation of behavioral parameters is a noninvasive way of detecting heat stress [45]. Previously CV has been used for the generation of cluster and unrest indices, developed respectively by [23] and [24], and have been applied as indicators of thermal comfort in commercial poultry production. The results obtained indicated that the unrest index could detect the agitation of poultry under different thermal conditions, with a significant decrease in the movement of birds under heat stress [24]. 

On the other hand, the cluster index revealed a significant difference in the clustering behavior of birds under conditions of comfort and heat stress. In addition, it identified behavioral differences between the heavy rearing breeds [23]. The unrest index was used to measure the walking ability of broilers with different gait scores [46]. Both indices have the potential to develop a remote-monitoring system to accurately detect differences in the behavior of birds raised in floor pens bedded with wood shavings. The application of these indices has not yet been explored in the rearing of broilers in enriched environments.

This study is a proof of concept to assess the use of the cluster and unrest indexes simultaneously to test the sensitivity and viability in order to evaluate the movement of broilers under different conditions such as heat stress and pen enrichment.

## 2. Materials and Methods

### 2.1. Description of the Controlled-Environment Chamber

**Figure 1:**
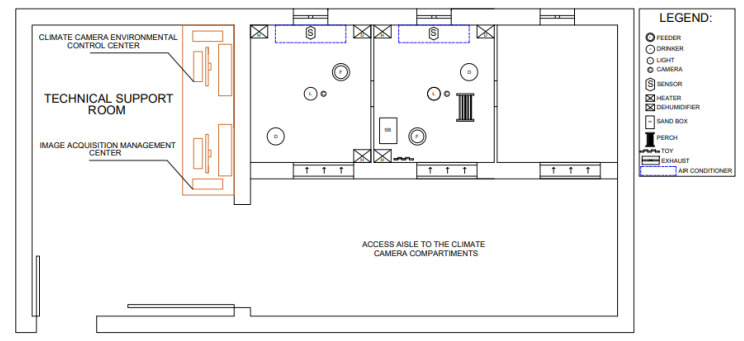
Plan view of the environmental chamber (adapted from [47]). Reprinted/adapted with permission from Ref. [47]. 2022, Daniella Jorge de Moura.

The controlled-temperature room has three compartments (C1, C2 and C3), measuring 1.6 × 1.4 × 3.0 m^3^. Only two compartments were used for the present study, and they were randomly selected. In each compartment, a manual tube-type feeder (Zatti^®^ Model number 181,528, Zatti Industry and Commerce, Coronel Freitas, Santa Catarina, Brazil), an automatic pendulum-type drinker (CASP^®^, Model pendular drinking automatic 2003, CASP, Amparo, São Paulo, Brazil), and a temperature and humidity sensor were installed close to the animals’ level, at a distance of 0.40 m from the floor. Each compartment had an air conditioner, two dehumidifiers, two heaters, a dimmable LED lamp to control light intensity (lx), and a video camera. Each compartment was accessed independently through a door (0.7 m wide × 2 m high). The computers responsible for managing the experimental units were installed in the support room (climate control and video recording). Figure 1 [47] shows the schematic with the respective positioning of all the equipment used in the controlled-temperature room and the technical-support room during the experimental period.

**Figure 2:**
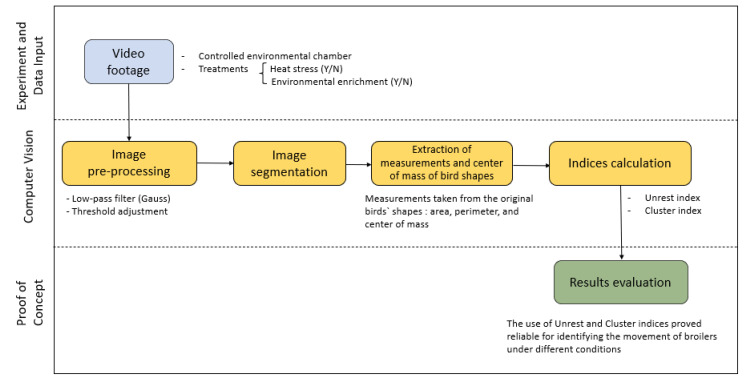
Diagram of data input and output of the tests.

The environmental-control center manages each compartment of the controlled-environment room using software developed in the Delphi programming language (version 6.0, Borland Software Co., Austin, TX, USA). The software allows measuring, processing, controlling, and recording continuously collected data. This system allows the user to check temperature, humidity, light intensity, and air renewal rates in real time. According to the established temperature and humidity values, the equipment is automatically activated (on and off). Relative humidity was programmed to remain at 60% continuously. Only the air temperature varied during the experimental period, with operating values recommended by the [48] of 23 °C for thermal comfort and 31 °C for heat-stress treatment. The particularities of the environmental-control system, equipment, operating limits, stability, and validation with broilers are presented in [47]. The steps of inputs, data collection and processing, experimental treatments, and outputs are represented in Figure 2 and explained in the sequence.

### 2.2. Image Acquisition

**Figure 3:**
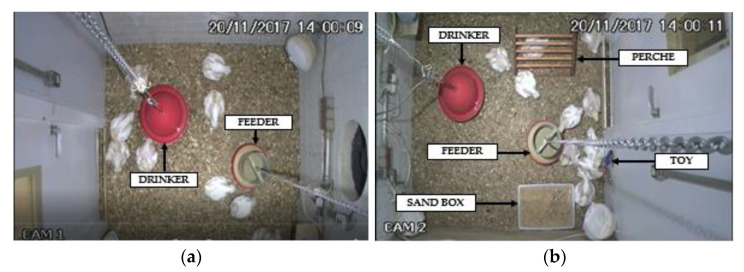
Top view of the unenriched (**a**) and enriched (**b**) compartment through the cameras installed on the ceiling of each compartment of the climate chamber.

For the animal-behavior monitoring data and further analysis, surveillance cameras (Intelbras^®^ VMD 3120 IR, Intelbras Corporation, São José, Santa Catarina, Brazil) with a resolution of 976 × 496 (H × V) and automatic activation of the infrared device in cases of low light were installed on the ceiling of the geometric center of each compartment. The two validation tests used recorded video images between noon and 18:00 h. Video recordings were automatically stored on an NVR video recorder (Intelbras^®^ Multi HD Serie 1000, 1080p, Intelbras Corporation, São José, Santa Catarina, Brazil). Figure 3 shows the areas observed for the unenriched (a) and enriched (b) compartments.

### 2.3. Birds and Husbandry

A total of thirty-day-old mixed-sex chicks of the Cobb^®^ strain were obtained from a commercial farm. Twenty chicks with similar weights and the same distribution of males and females were selected in two treatments, each containing ten birds (without environmental enrichment and with environmental enrichment). The compartments and animals were randomly assigned to assign the treatments on the first day of housing in the controlled-environment chamber.

Both compartments were kept without environmental enrichment during the first three days of adaptation in the climatic chamber. The compartment selected as “enriched” was provided with colored plastic rings suspended by a string, a plastic box containing fine sand, and a wooden perch. According to previous literature, the enrichment was selected to positively affect the birds’ natural behaviors (perching, pecking, and dust bathing) [13,49,50]. 

A bell drinker and feeder were placed in each compartment. Water and commercial feed, based on corn and soybean meal, were provided ad libitum throughout the rearing period. The supply of commercial feed and water to the birds was ad libitum throughout the experimental period and followed the nutritional recommendations of the breeder’s manual [48]. Once a day, the offered ration was weighed and manually inserted into the tube feeder. The automatic water-supply system used a tubular drinking fountain with height adjustment. The floor was covered with shavings bedding (0.05 m). We adopted the breeding-company-recommended period of light (24 h of light until the seventh day and increasing 1 h of darkness every two days). On the 14th day of growth, the birds remained exposed to 20 h of light and 4 h of darkness from 21:00–01:00 until the end of the experiment (42nd day of growth). We also adopted the breeding manual, so the broilers were kept in thermoneutrality conditions during the first to the twentieth day of growth [48].

**Figure 4:**
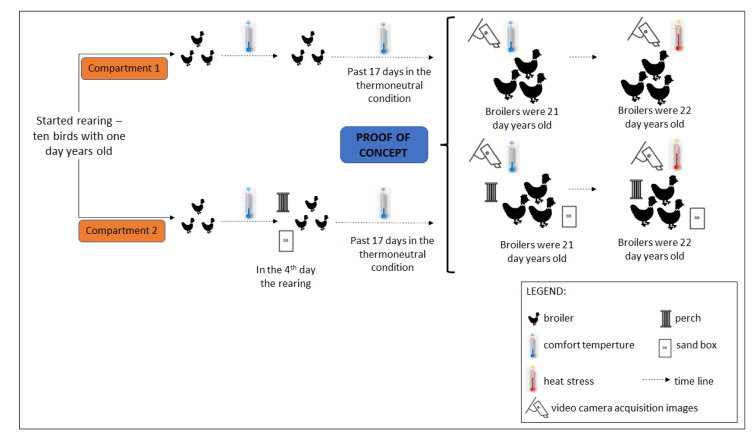
Diagram of the methodology used for proof-of-concept tests.

The acquisition of video images was performed automatically for seven consecutive hours from noon to 18:00 h for the two consecutive days of analysis. According to a previous study [5], broilers’ activity patterns were similar for three weeks throughout the day. Such assumption allowed us to validate the analysis of broiler movement through the cluster and unrest indexes in two days for experimental conditions in the controlled-environment room, characterizing the present study as a proof of concept. The age of 21 days is when the heat stress starts to impair productive performance (decrease in feed consumption and weight gain) and negatively challenge animal metabolism and immunity [43,51,52]. Our experimental tests were precisely at the age of 21 and 22 days under thermoneutrality and heat-stress conditions, respectively, for both treatments. Heat stress was tested for one day, with the chickens at 22 days of age, with the birds being kept in thermal comfort during the previous housing days. Figure 4 illustrates the diagram of activities used in data collection for proof-of-concept validation.

The relative humidity remained within what was recommended in the breeders’ manual [48] during the experiment. A previous commissioning study of the controlled-environment-room operation [47] allowed one hour to reach the heat-stress temperature condition (31 °C) and maintain the system. For this reason, the control setup started at noon.

### 2.4. Video Analysis

The efficiency of two comfort indexes based on group behavior was verified. The calculation of these indexes is based on information extracted from images recorded through image-analysis techniques. This proof of concept evaluated the extraction of information and the calculation of indexes by a computer-vision system.

Videos were analyzed at the frequency of one frame per second (fps). Considering that there was no effect of the compartments, we used a completely randomized design in a split-plot scheme in time, in which we tested two factors: (1) temperature (comfort or heat stress), as the main factor; and (2) environmental enrichment (present or absent), as the secondary factor. The seven hours of recording analyzed were divided into 14 blocks of time (30 min each block, which corresponds to the analysis of 25,200 frames per condition (temperature vs. environment), totaling 100,800 frames in the experimental period. 

The images were processed frame by frame, initially using low-pass filters to smooth out image noises such as feathers on the bedding and wood shavings on the birds. After segmentation, mathematical morphology techniques were applied to fill holes and exclude the remaining noise. The group behavior of chickens was measured using the cluster index [23] and the unrest index [24], described in Equations (1) and (2).

(1) $${\mathrm{Cluster}\;\mathrm{Index}}_{i}=\frac{2\times \bar{A}\times \sqrt{h^{2}+w^{2}}}{\bar{P}\times \bar{D}\times n_{A}}-1$$

where ${\mathrm{Cluster}\;\mathrm{Index}}_{\left(i\right)}$ is the cluster index of the birds observed in the ith frame of the video; $\bar{A}$ and $\bar{P\;}$ are the average area and perimeter (in pixels) of the shapes observed in the frame, respectively; $\bar{D}$ is the average distance between the centers of mass of the shapes in the scene; $n_{A}$ are the number of clusters; and h and w correspond to the height and width (in pixels) of the cropped image.

(2) $${\mathrm{Unrest}\;\mathrm{Index}}_{\left(i,i-1\right)}=k.\max\left\{\mathrm{dH}\left(F_{\left(i\right)},F_{\left(i-1\right)}\right),\;\mathrm{dH}\left(F_{\left(i-1\right)},F_{\left(i\right)}\right)\right\}$$

where ${\mathrm{Unrest}\;\mathrm{Index}}_{\left(i,\;i-1\right)}$ is the unrest index (cm) of the birds between two frames recorded with 1 (one) second difference; i is the position of the frame in the video; $F_{\left(i\right)}$ is the current frame; $F_{\left(i-1\right)}$ is the previous frame; dH is the Hausdorff distance [53] between birds from one frame to another; and k is the proportionality factor calculated by Equation (3).

(3) $$k=\frac{2H\tan\left(\alpha /2\right)}{w}$$

where k is the proportionality factor; *H* is the height (cm) of the installed camera concerning the floor; α is the opening angle of the camera lens; and w is the length (pixels) of the CCD sensor, which corresponds to the length of the largest measurement of the frame captured by the camera. The video capture rate was 30 fps, but a frequency of 1 fps was adopted as the most adequate for image analysis, considering the birds’ movement speed.

Cluster and unrest indexes were calculated frame by frame. In this way, the values obtained for each plot correspond to an average referring to 1800 images. The data were explored by graphs of the indexes calculated in the simultaneous application observation time, verifying possible interactions and differences in the crowding and unrest behaviors between the enrichment and temperature treatments evaluated. We applied the ANOVA with repeated measures, followed by the Tukey mean test to confirm the differences in the birds’ crowding and movement behavior between the evaluated environmental treatments.

## 3. Results and Discussion

**Figure 5:**
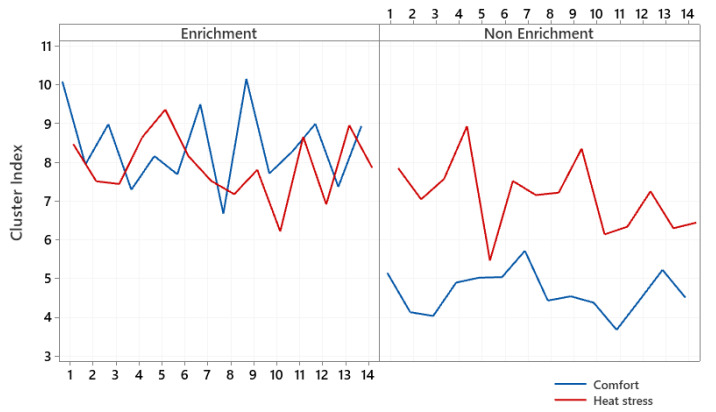
Cluster index of the group of broilers housed in enriched and nonenriched environments under the effect of comfort and heat stress.

Figure 5 corresponds to the results obtained for the cluster index (crowding) from noon to 18:00 in the group of broilers housed in an enriched and nonenriched environment, subjected to thermal conditions of neutrality and heat stress. Each point on the graph represents a repetition of a subdivision plot in time (total 14). Each plot corresponds to the analysis average of 1800 frames/video for generating the cluster index for the treatments.

It can be seen from Figure 5 that the cluster index detected in the enriched environment is similar between the conditions of thermoneutrality and heat stress. However, the highest peaks occurred in thermoneutrality and the lowest in heat stress. This observation of trend analysis means that the ambient temperature above the comfort limit was not enough to change the crowding pattern of broilers aged 21 days reared in enriched environments. Therefore, the isolated analysis of this index cannot be considered an indicator of heat stress for broilers raised in enriched environments. We observed significantly lower crowding rates in the comfort temperature and nonenriched environment than the enriched-compartment results.

**Figure 6:**
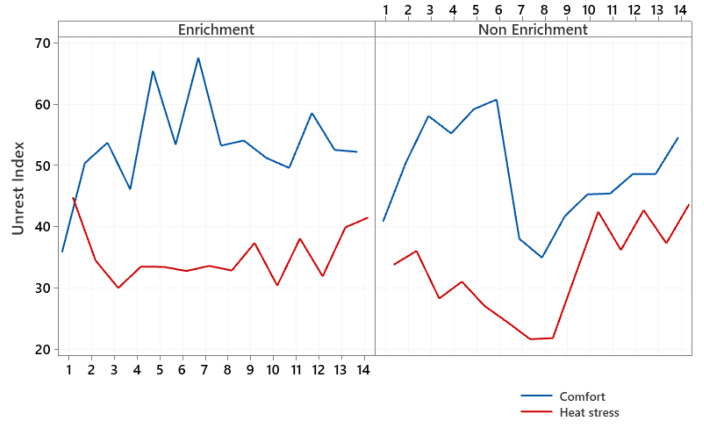
Unrest index of the group of broilers housed in enriched and nonenriched environments under the effect of comfort and heat stress.

Figure 6 illustrates the unrest index for the treatments from noon to 18:00 h. Each point of the graph represents a repetition of the subdivision plot in time (total 14). Each plot corresponds to the average of 1800 frames/video analysis for the generation of the unrest index of the treatments.

During the evaluated period, a tendency for the unrest index to be higher under thermoneutrality conditions is observed, regardless of the environmental enrichment. Furthermore, heat stress reduces the movement of animals, with more significant losses for the nonenriched treatment. Table 1 shows the differences observed in the behavior of gathering and movement of the birds, where it is observed that the environmental enrichment promoted more movement of the birds both under conditions of comfort (52.74 vs. 48.71) and thermal stress (35.38 vs. 32.81), which confirm previous graphical analyses. However, heat stress is a limiting factor for the movement of animals, reducing the positive potential of the presence of environmental enrichment. Environmental enrichment provided higher crowding rates both in comfort (8.42 vs. 4.66) and in heat stress (7.91 vs. 7.11), and in an enriched environment, birds under heat stress crowded more than birds raised in nonenriched environments (4.66 vs. 7.11). 

Environmental enrichment increased broilers’ unrest (movement) index from fast and slow-growing strains under thermoneutrality conditions, confirming our findings [32]. Exposure to high temperatures above the thermal comfort zone is challenging for birds housed in complex environments. Broilers raised in enriched environments from 1-day-old exposed to stress conditions at 22 days of age (heat, noise, and containment in a crate) showed heat stress as the worst adverse condition [54].

Environmental enrichment benefits, especially perches and litter boxes, have been extensively studied [2,4,7,9,10,11,50]. In the present study, we noted behavioral changes in the group compatible with these benefits, suggesting that the proposed computer vision based on cluster and unrest indexes can be safely used for these assessments.

**Figure 7:**
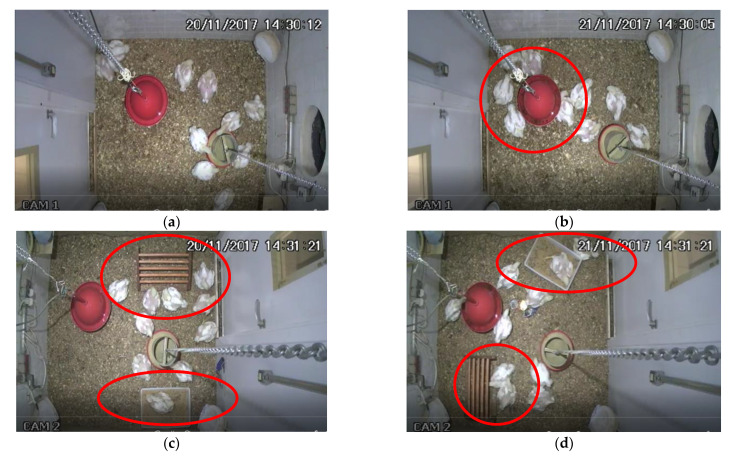
Frames evaluated are (**a**) nonenriched environment in comfort, (**b**) nonenriched environment under heat stress, (**c**) environment enriched in comfort, and (**d**) enriched environment under heat stress.

Figure 5 shows the crowding behavior of broilers housed in enriched and nonenriched environments at the different temperatures tested. Results of the present study indicated that complex environments favored the crowding of the group of broilers under heat stress. Visually reviewing the videos, we observed that birds under heat stress conditions clustered around enrichment objects, indicating that environmental enrichment can minimize the negative effect of heat stress on birds. Figure 7 illustrates the frames for comfort (a and c) and heat stress (b and d) conditions in enriched (a and b) and nonenriched (c and d) environments.

When analyzing the positioning of the animals in the video images, it was observed that broilers have different distribution patterns depending on the complexity of the environment (enriched versus nonenriched). Note that the birds are better distributed throughout the compartment for the environment without environmental enrichment in the comfort treatment (Figure 7a). However, in the heat-stress condition (Figure 7b), birds crowded near the drinker to benefit from the microclimate close to the water, which resulted in higher crowding rates [23,55]. The behaviors of remaining seated birds—increased water consumption, spreading wings, increased respiratory rate, and panting—are favored to dissipate excess heat [55,56,57,58].

In our study and previous work, bird distribution inside the pen appears to be highly related to the location of food and water [59]. Environmental enrichment also alters the distribution pattern of birds, with a higher prevalence of agglomeration close to enrichment objects, both in small-group experiments [32,60,61] and at a commercial scale [2,8,62]. Cornetto and Estevez [60] observed that the birds were forced to occupy the central region earlier in groups of larger sizes than for smaller group sizes. Slow-growing broilers used environmental-enrichment objects more frequently when compared to fast-growing broilers [32]. These findings reinforce the differences among housed flocks and the importance of recalibrating the CV system to each housing situation (group size, strains, age, and housing conditions).

The CV could simultaneously apply the Cluster and Unrest indexes to monitor the movement of the group of broilers under different environmental conditions, indicating the possible differences in the environmental conditions. The authors suggest that more research should be conducted to evaluate the potential positive impact of environmental enrichment in poultry production. The complexity of the environment is a factor to be considered in creating alert systems for detecting heat stress in broiler production.

## 4. Conclusions

The cluster and unrest indexes calculated from videos analyzed by computer-vision techniques allowed us to simultaneously evaluate the movement of broilers raised in different environments and detect variations that allowed us to estimate the level of wellbeing. We recommend that the indexes be used to evaluate the movement and agglomeration of broiler flocks in environments with different enrichment levels to evaluate the improvement of wellbeing. In large groups, birds’ movement and grouping patterns may differ; therefore, the CV system and indices will need to be recalibrated. The use of CV to assist with monitoring can assist caregivers during the rearing of broiler chickens.

## Figures and Tables

**Table 1:** Results of the Tukey test (*p* < 0.05) for the cluster and unrest indexes in the combined conditions of temperature and environmental enrichment found in the proof-of-concept experiment.

Indexes	Temperature	Environment	
		Enriched	Nonenriched
Cluster	Comfort	8.42	4.66
	Heat stress	7.91	7.11
Unrest	Comfort	52.74	48.71
	Heat stress	35.38	32.81

## Data Availability

Data will be available upon request.
